# The PHR proteins: intracellular signaling hubs in neuronal development and axon degeneration

**DOI:** 10.1186/s13064-016-0063-0

**Published:** 2016-03-23

**Authors:** Brock Grill, Rodney K. Murphey, Melissa A. Borgen

**Affiliations:** Department of Neuroscience, The Scripps Research Institute, Scripps Florida, Jupiter, FL 33458 USA; Department of Biological Sciences, Florida Atlantic University, Jupiter, FL 33458 USA

**Keywords:** Synapse formation, Axon termination, Axon guidance, Axon degeneration, Phr1, Pam, MYCBP2, Highwire, RPM-1, PHR protein

## Abstract

During development, a coordinated and integrated series of events must be accomplished in order to generate functional neural circuits. Axons must navigate toward target cells, build synaptic connections, and terminate outgrowth. The PHR proteins (consisting of mammalian Phr1/MYCBP2, Drosophila Highwire and *C. elegans* RPM-1) function in each of these events in development. Here, we review PHR function across species, as well as the myriad of signaling pathways PHR proteins regulate. These findings collectively suggest that the PHR proteins are intracellular signaling hubs, a concept we explore in depth. Consistent with prominent developmental functions, genetic links have begun to emerge between PHR signaling networks and neurodevelopmental disorders, such as autism, schizophrenia and intellectual disability. Finally, we discuss the recent and important finding that PHR proteins regulate axon degeneration, which has further heightened interest in this fascinating group of molecules.

## Background

Construction of neural circuitry relies upon axons accomplishing several important developmental tasks. Axons must extend and correctly navigate to a target cell, form functional synaptic connections in the correct location and with the correct density, and terminate outgrowth in a temporally and spatially precise fashion. A single family of proteins called PHR proteins, named after human Protein Associated with Myc (PAM), Drosophila Highwire and *C. elegans* Regulator of Presynaptic Morphology 1 (RPM-1), function in all these key steps in the development of an axon. Here, we review genetic, proteomic and biochemical findings that establish the PHR proteins as functionally conserved regulators of nervous system development from *C. elegans* through mammals. We also discuss how accumulated evidence has begun to support two major concepts regarding PHR proteins and nervous system construction. The first is that the PHR proteins function as intracellular signaling hubs that regulate numerous downstream pathways. The second is the “coordinator hypothesis”, our postulate that the PHR proteins are likely to coordinate different events in axon development. Finally, we explore the emerging and important role of PHR proteins in axon degeneration.

### The PHR proteins: conserved regulators of neuronal development

The conserved PHR protein family consists of human PAM (also called MYCBP2), mouse Phr1, zebrafish Esrom/Phr1, Drosophila Highwire, and *C. elegans* RPM-1 [1–3]. PAM was the first PHR protein discovered as part of a phage screen for Myc binding proteins [4]. Initial functional insight emerged when independent forward genetic screens revealed that Drosophila Highwire and *C. elegans* RPM-1 regulate both synapse formation and axon termination [5–7]. Subsequent studies in fish and mice showed that PHR proteins are conserved regulators of axon and synapse development [8–11]. These functional observations are consistent with broad expression of the PHR proteins in the developing and adult nervous system [6, 7, 9, 12–15]. Below, we explore PHR protein function in the developing nervous system across species. Commentary on the mechanisms of how PHR proteins regulate development of the nervous system follows.

#### Synapse formation

Highwire has been studied extensively in the developing larval nervous system. Loss of *highwire* function results in increased numbers of abnormally small synaptic boutons at the neuromuscular junction (NMJ) [5]. Defects in synapse morphology are accompanied by increased axon length, increased axon branching, and impaired presynaptic transmission. Highwire function is required exclusively in the motor neurons, as opposed to surrounding muscles, for proper synapse formation and function. In adult *highwire* mutants, neuronal function is also impaired in the giant fiber system (Borgen and Murphey, unpublished observation). Highwire functions both cell autonomously and in the surrounding midline glia to regulate giant fiber function.

Formation of NMJs is also abnormal in *rpm-1* null worms [6, 16]. Rather than the expanded number of synapses observed in *highwire* mutants, *rpm-1* mutants have reduced synapse number. This results from large axon segments that lack synapses despite smaller regions of the motor axon that have increased synaptic density. Synaptic transmission in *rpm-1* mutants has not been tested with electrophysiology, but pharmacological results suggest *rpm-1* mutants could have modest defects in GABAergic motor neuron function at the NMJ [17]. Given these pharmacological results with *rpm-1* mutants and synaptic transmission defects in *highwire* mutants, it seems likely that *rpm-1* mutants will have abnormal synaptic transmission. Importantly, neuron-neuron synaptic connections between the mechanosensory neurons and interneurons are impaired in *rpm-1* mutants [7]. Like Highwire, RPM-1 functions cell autonomously in motor neurons and mechanosensory neurons to regulate synapse formation.

It is curious that loss of PHR protein function results in different synapse morphology defects in worm and fly motor neurons. This could be rooted in the distinct anatomy of how fly and worm motor neurons form synapses: worms forming synapses *en passant* along the length of muscles, and flies having terminal axon branches that innervate muscles. Alternatively, the dense packing of presynaptic terminals in portions of the axon in *rpm-1* mutants could be thought of as regions where expanded synapse formation is occurring. In this case, it could be argued that a portion of the phenotype in *rpm-1* mutants is similar to what is observed in flies. However, even with this interpretation there are clearly regions of the motor axon lacking synaptic connectivity, which differs from the phenotype in *highwire* mutants. Nonetheless, despite phenotypic differences between *rpm-1* and *highwire* mutants, it is clear that PHR proteins regulate synapse formation in invertebrates.

Mice that lack *Phr1* function die shortly after birth due to respiratory distress from impaired innervation of the diaphragm [9–11, 18]. Two mice with *Phr1* impairment have been tested for synapse defects: mice with a small, multi-gene deletion that includes *Phr1* (*Phr1*^*Df*^) [9]; and gene-targeted mice that have an in-frame deletion of exons 8 and 9 (*Phr1*^Δ*8,9*^) [10]. In both cases, motor neurons have reduced numbers of NMJs, and have abnormal orphan presynaptic terminals that lack postsynaptic connections. Like invertebrates, Phr1 functions cell autonomously in the motor neurons to regulate synapse formation [10].

These findings from worms, flies, and mammals show that PHR proteins are conserved regulators of synapse formation in motor neurons. However, several questions remain unresolved. For example, do synapse formation defects caused by loss of PHR protein function arise from impaired synapse assembly, maturation, or stability? Do vertebrate PHR proteins regulate synapse formation in the central nervous system?

#### Axon termination

Over the course of development, axons extend, reorient growth in response to guidance cues, and form synapses. Eventually, growth stops via a process we refer to as axon termination. Compared to axon guidance or synapse formation, we know relatively little about how axon termination is regulated. The PHR proteins have emerged as important, conserved regulators of axon termination. In *C. elegans rpm-1* mutants, many types of neurons have severe axon termination defects, in which axons fail to stop at the anatomical location where extension normally halts. These neurons include: mechanosensory neurons (ALM and PLM) [7, 19], motor neurons (DD and VD) [20], and pharyngeal neurons (M1) [21]. RPM-1 functions cell autonomously in the mechanosensory and motor neurons to regulate axon termination. Importantly, *rpm-1* mutants show defects in both axon termination and synapse formation in single motor neurons or mechanosensory neurons [7, 20]. Thus, RPM-1 regulates both axon termination and synapse formation in individual neurons.

Relatively little is known about extracellular cues that regulate axon termination, but progress has been made with the PLM mechanosensory neurons of worms. Axon termination defects caused by *rpm-1* (lf) are suppressed by *unc-6/Netrin* or *slt-1/Slit* [22]. This suggests that Netrin and Slit are the attractive guidance cues that facilitate abnormal PLM axon overgrowth once the axon termination signal is impaired in *rpm-1* mutants. However, the extracellular cues that trigger PLM axon termination would not be expected to suppress defects in *rpm-1* mutants. Rather, triggers of termination would be expected to act in the same pathway or a parallel enhancer pathway to *rpm-1*. Consistent with this model, a recent study showed that multiple Wnts, but primarily LIN-44, regulate PLM axon termination [23]. In the future, it will be important to determine if other guidance cues function with Wnts to regulate axon termination, and whether any of these cues function upstream of RPM-1.

Several observations suggest that axon termination could be impaired with loss of Highwire and Phr1 function. In larval flies, the motor neurons of *highwire* mutants have dramatically increased axon branch length [5]. This is often considered part of a synaptic overgrowth phenotype, but could reflect failed axon termination. The class IV dendritic arborization (C4da) larval sensory neurons of *highwire* mutants have overgrown axon terminals, which might also reflect axon termination defects [24]. It is important to note that while we prefer to use the term axon termination, all the phenotypes of this nature could certainly result from a failure to restrict axon growth. Whether this reflects semantics, or an important mechanistic distinction, will require a much more thorough knowledge of the molecular, cellular and developmental mechanisms at play.

The sensory neurons from two different mice that lack *Phr1* function have axon termination defects. Sensory axons in the skin fail to terminate correctly and are heavily overextended in *Phr1*^*Df*^ mice and *Magellan* mutant mice (*Phr1*^*Mag*^), which have a premature stop codon that deletes the C-terminal third of Phr1 [9, 11]. The axons of sensory neurons are bifurcated and also have precise axon termination sites in the spinal cord [25–27]. These termination sites in the central nervous system are enlarged in *Phr1*^Δ*8,9*^ mice suggesting that axon termination could be impaired [28].

There is also evidence of defective axon termination in the visual system of fish and mice lacking Phr1 function. In zebrafish *esrom/Phr1* mutants, retinal axons primarily have axon guidance defects, which we discuss in the subsequent section. However, those retinal axons that complete the guidance program successfully, though small in number, have an enlarged field of axon innervation suggesting axon termination is defective [29]. Likewise, retinal axons targeting the superior colliculus have enlarged termination zones in *Phr1*^Δ*8,9*^ mice, which suggests that Phr1 regulates axon termination in a subset of retinal ganglion cells [30].

These findings indicate that the PHR proteins are conserved regulators of axon termination, in the peripheral and central nervous system. Nonetheless, an important question lingers. What are the underlying cellular and developmental defects that result in failed axon termination in animals lacking PHR protein function?

#### Axon guidance

Axon extension and guidance are developmental events that precede axon termination. A role for PHR proteins in axon guidance was first discovered using genetic screens in zebrafish [8, 29, 31]. In fish, retinal axons normally extend, arborize and innervate the posterior optic tectum. *esrom* mutants have impaired retinal axon guidance, which leads to premature anterior arborization and failed innervation of the optic tectum [8]. Retinal axons of *esrom* mutants also have reduced midline crossing [29, 31]. Similarly, loss of Phr1 results in defective retinal axon targeting in mice [32]. *Phr1*^Δ*8,9*^ mice have both abnormal number, shape, and location of retinal axon projections on the dorsal-lateral geniculate nucleus (dLGN). Similar to Esrom, Phr1 functions cell autonomously in retinal axons to regulate axon targeting to the dLGN.

The function of Phr1 in axon guidance is not limited to the visual system. *Phr1*^Δ*8,9*^ mice have a plethora of axon guidance defects in the brain indicating that Phr1 has a broad and important role in regulating construction of neural connectivity. These defects include: 1) loss of the anterior commissure and internal capsule; 2) reduction in the width of the corpus callosum; 3) reduced numbers of cortical axons; 4) loss of thalamocortical projections; and 5) reduced sensory neuron innervation of the olfactory bulb [10, 33]. Interestingly, defects in axon guidance in the brain arise from Phr1 functioning cell autonomously in neurons, as well as non-cell autonomously [10]. For example, Phr1 functions cell autonomously in cortical neurons to regulate axon guidance through the corpus callosum. In contrast, defects in axon guidance that result in reduced cortical axon extension through the internal capsule are the result of non-cell autonomous Phr1 function. The guidance defects in *Phr1*^Δ*8,9*^ mice often reflect a problem with stalling at guidance choice point boundaries, as opposed to impaired axon extension. This reflects a conserved theme for PHR proteins in axon guidance, as axons stall at boundaries in the brains of *esrom* mutant fish resulting in loss of the habenular commissure [34].

Phr1 is also an important regulator of axon guidance in the peripheral nervous system. Extension and branching of the phrenic nerve is greatly reduced in the diaphragms of *Phr1*^*Df*^ and *Phr1*^Δ*8,9*^ mice [9, 10]. *Phr1*^*Mag*^ mice were initially identified in a mutagenesis screen for genes that regulate motor axon guidance [11]. These animals have axon guidance defects at points where motor axons exit from the spine. Further, motor axon bundles have severely impaired guidance in the hind limb. Abnormal axon guidance in mice lacking Phr1 is consistent with the observation that cultured motor and sensory neurons from *Phr1*^*Mag*^ mutant mice have abnormal growth cone morphology and dynamics [11].

While vertebrate PHR proteins are important regulators of axon guidance in the central and peripheral nervous system, invertebrate PHR proteins play a much more limited role in axon guidance. In *highwire* mutants, impaired axon guidance results in failed separation of axon lobes in the Mushroom Body [35]. Like some guidance events regulated by Phr1, Highwire functions non-cell autonomously to regulate Mushroom Body axon guidance. RPM-1 regulates axon guidance in the AVM mechanosensory neuron and the cholinergic motor neurons of *C. elegans* [22]. However, RPM-1 is not a primary player in these guidance events, as a *Netrin* (lf) or *Sli*t (lf) sensitizing background is required to reveal RPM-1 effects on guidance. Similarly, a *syd-2/liprin* (lf) sensitizing background is required to reveal a role for RPM-1 in regulating axon extension in GABAergic motor neurons [20]. These findings indicate that invertebrate PHR proteins first emerge evolutionarily as relatively minor regulators of axon guidance, but take on greatly expanded roles in vertebrate axon guidance in both the peripheral and central nervous system.

#### Axon pruning

Recent work in the developing pupal fly has shown that Highwire regulates pruning of giant fiber axons. In adult *highwire* mutants, giant fiber axons exhibit an ectopic branch that grows past the synaptic target [36]. Careful analysis in pupae showed that giant fiber axons normally overshoot the desired synaptic target and are pruned back (Borgen and Murphey, unpublished observation). The persistence of overextended giant fiber axons in *Highwire* mutants suggests that axon pruning is impaired. Similar to the giant fiber axons, mammalian motor neurons undergo extensive pruning of terminal axon branches to eliminate synapses [37, 38]. While the presence of overextended orphan presynaptic arbors in motor neurons lacking Phr1 could reflect axon termination defects, this defect might also result from impaired axon pruning [9, 10]. It will be interesting to see what other pruning events are affected by PHR proteins.

#### Postsynaptic and dendritic function

PHR proteins do not function solely in a presynaptic and axonal capacity. The first example of this was the discovery that RPM-1 regulates glutamate receptor endocytosis at the postsynaptic terminal of interneurons in *C. elegans* [39]. Likewise, Highwire regulates not only axon termination in fly larval sensory neurons, but also dendritic arborization [40]. As noted earlier, larval sensory neurons in *highwire* mutants have axon termination defects, in which axons overextend. In contrast, dendritic arbors are reduced in length and branch number. Thus, PHR proteins can have opposing effects on axon termination and dendrite extension in a single neuron.

#### PHR protein expression and localization in neurons

The function of PHR proteins in synapse and axon development is consistent with the subcellular localization of these molecules in neurons. Highwire and RPM-1 are localized to the presynaptic terminal [6, 12, 13, 19], which is consistent with PHR proteins functioning in motor neurons to regulate synapse formation and function. RPM-1 is localized to a perisynaptic region of the presynaptic terminal, which is adjacent to the synaptic vesicles and the active zone. The molecular composition of the perisynaptic region where RPM-1 is localized remains minimally characterized. In worms, RPM-1 is also highly concentrated at the mature axon tip of both motor neurons and mechanosensory neurons [12, 19, 20]. These localization patterns are consistent with RPM-1 regulating both axon termination and synapse formation in single neurons. Interestingly, the conserved PHR1 domain of RPM-1 is necessary and sufficient for localization of RPM-1 to both the presynaptic terminal and the axon tip [12]. While the mechanism of how RPM-1 is localized remains unknown, the crystal structure of the PHR1 domain indicates that PHR1 has a relatively flat, conserved surface that is likely to mediate protein-protein interactions [41]. The subcellular localization of RPM-1 prompts several interesting questions. What is the temporal relationship between RPM-1 localized at the terminated axon tip and the presynaptic terminal? Does localization to one compartment precede the other? Is RPM-1 deposited before, during or after synapse assembly occurs?

The anatomy of fly larval motor neurons makes it difficult to distinguish Highwire at the presynaptic terminal from Highwire that could be concentrated at the mature axon tip. While the localization of endogenous Highwire remains unknown, transgenic Highwire is present at presynaptic boutons [13]. It is unclear if Highwire is localized, or enriched at terminal boutons on the end of motor axon branches. Addressing this could prove helpful in supporting or refuting the idea that increased axon length in *highwire* mutants is the result of defective axon termination, as opposed to being the result of unrestricted growth. Transgenic Highwire also localizes to vesicles/puncta in motor axons and cell bodies [42].

Vertebrate PHR proteins are more broadly localized. In cultured cortical and retinal neurons, Phr1 and Esrom are localized to puncta throughout the axon and dendrites, including synaptic puncta [8, 43]. Phr1 is also detected in growth cones of cortical neurons. In contrast, Phr1 is localized throughout the axon but is excluded from growth cones in motor and sensory axons [11]. These differing results suggest that Phr1 localization in the growth cone could shift significantly with the developmental state of the axon, or in different types of neurons. For example, motor and sensory axons might need more rigid exclusion of Phr1 from the growth cone in order to facilitate axon extension across the large distances they must traverse in the periphery. Nonetheless, Phr1 localization in or around axonal growth cones is consistent with Phr1 regulating growth cone morphology and dynamics, as well as axon guidance [11, 44]. Phr1 expression and localization has not been explored in glia, but this could be useful since Phr1 regulates axon guidance in the brain, in part, through non-cell autonomous mechanisms.

#### Behavior

Given the importance of PHR proteins in axon and synapse development, one would expect these molecules to impact behavior. *highwire* was originally named after the mild walking defects that are present in adult *highwire* mutants [5]. However, the neuronal defects that lead to this phenotype remain unknown. Given that larval motor neurons have abnormal synapse formation and function, it is plausible walking defects might result from impaired synapse development in the adult nervous system. Alternatively, postdevelopmental functions of Highwire in adult flies could be responsible for walking defects. Although the motor neurons of *highwire* mutant larvae have strong defects in synapse formation and function, no defects in the locomotion of *highwire* larvae have been described to our knowledge.

Quantitative behavioral analysis of adult flies showed that *highwire* mutants have abnormalities in long-term memory formation [45]. *highwire* mutants accumulate long-term memories in response to aversive odors with dramatically fewer trials than wild-type animals. This suggests that Highwire functions as a negative regulator of aversive memory gating. Interestingly, Highwire functions post-developmentally to regulate long-term aversive memory. This suggests that the axon guidance defects in the Mushroom Body neurons of *highwire* mutants are not severe enough to prevent formation of olfactory memories, and are not responsible for abnormal gating of long-term memory.

*rpm-1* mutants were initially found to have normal locomotion and normal gentle touch sensation. This occurs in spite of the fact that axon and synapse development is impaired in the neurons that mediate these behaviors [6, 7]. With loss of both *rpm-1* and regulators of active zone assembly, such as *syd-2/liprin* or *syd-1*, noticeable behavioral defects emerge, in particular strongly impaired locomotion [16, 46]. However, impaired locomotion is accompanied by enhanced synapse formation defects in the inhibitory and excitatory motor neurons [46]. Recently, more sensitive quantitative analysis uncovered mild defects in spontaneous locomotion in *rpm-1* mutants [47]. This is likely to represent the behavioral outcome of impaired synapse formation in the motor neurons of *rpm-1* mutants. More importantly, *rpm-1* mutants have extremely severe defects in behaviors that require plasticity or protracted circuit function, such as short-term learning. Temporal rescue experiments indicate that short-term learning defects in *rpm-1* mutants are not a consequence of adult RPM-1 function, but rather a consequence of developmental abnormalities caused by loss of RPM-1 function. These findings suggest that RPM-1 function in neuronal development has relatively minor impacts on innate behavior, but dramatic effects on plastic behavior, such as short-term learning.

Lethality caused by constitutive loss of Phr1 or Esrom has limited behavioral analysis in vertebrates. The severe axon guidance defects in the brains of *Phr1*^Δ*8,9*^ mice suggest that bypassing lethality with brain-specific ablation of *Phr1*^Δ*8,9*^ would still result in major behavioral deficits. However, because Highwire regulates long-term aversive memory in adult flies, it might be worthwhile to ablate *Phr1*^Δ*8,9*^ in the brains of adult mice after the developmental program has been completed. Further, investigating different behavioral outcomes after ablating *Phr1* in subsets of neurons could prove informative.

### PHR proteins as intracellular signaling hubs

As discussed above, PHR proteins have conserved functions in synapse formation, axon guidance, and axon termination. Over the past decade, a combination of genetic, proteomic and biochemical experiments have greatly expanded our understanding of PHR mechanisms of action. Below we discuss this diverse range of signaling activities, which are central to our proposition that PHR proteins function as intracellular signaling hubs.

#### Regulation of p38 MAP kinase, JNK MAP kinase, and TSC signaling

**Fig. 1:**
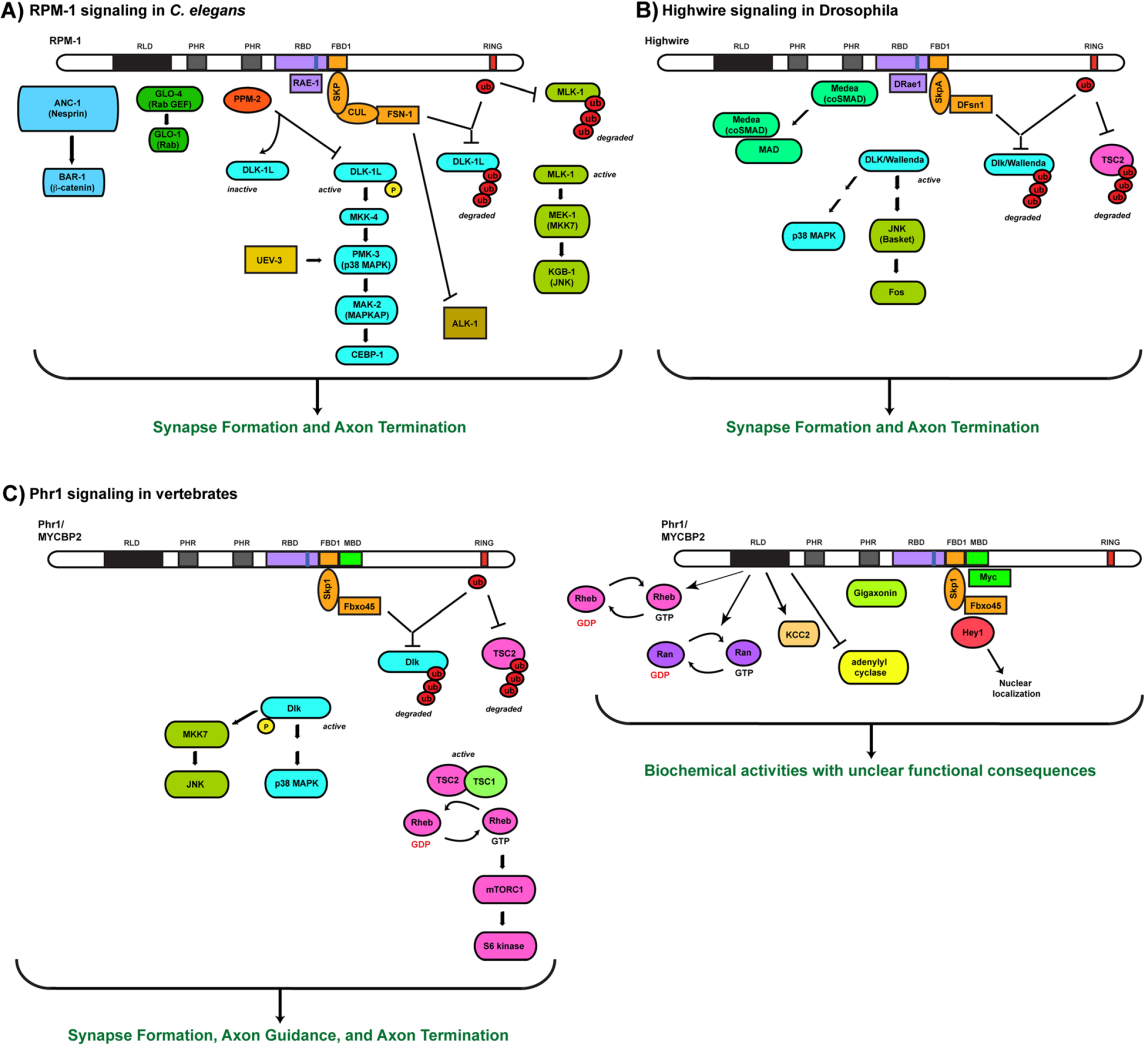
Overview of PHR protein signaling in worms, flies and vertebrates. PHR proteins function as intracellular signaling hubs that regulate numerous signaling pathways to control neuronal development. Molecules that bind to the PHR proteins are shown adjacent to (**a**) RPM-1, (**b**) Highwire, and (**c**) Phr1. For example, RPM-1 binds to ANC-1/Nesprin, GLO-4, PPM-2, RAE-1, and the FSN-1 complex. Direct protein interactions with RPM-1 are shown in direct contact and protein interactions that could be direct or indirect are shown with open space. The location of binding on PHR proteins is arbitrarily assigned for all proteins except RAE-1, the FSN-1/Fbxo45 complex, Myc, adenylate cyclase, Rheb, and Ran. GLO-4 binds to a large N-terminal portion of RPM-1, but this interaction has not been more extensively mapped. To date, FBD1 has only been tested for binding to FSN-1. Our diagram proposes that FBD1 is potentially the site where Skp anchors FSN-1/Fbxo45 on the PHR proteins. However, it is also possible FBD1 binds directly to FSN-1, and another site in the PHR proteins binds to Skp proteins. Note, several signaling pathways that function downstream of the PHR proteins have not been explored across model systems. FSN-1/Fbxo45, DLK-1/Dlk, JNK, p38 MAPK, and RAE-1 are conserved signaling mechanisms that mediate PHR protein function. Conserved protein domains in the PHR proteins are highlighted: RCC1-like GEF domain (RLD), PHR family specific domains (PHR), RAE-1 binding domain (RBD), FSN-1 binding domain 1 (FBD1), Myc binding domain (MBD) and RING-H2 ubiquitin ligase domain (RING)

The first foray into understanding how the PHR proteins regulate synapse formation was led by the Jin and Diantonio labs [16, 48]. A predicted RING-H2 ubiquitin ligase domain in the PHR proteins [5–7] and genetic enhancer effects with the deubiquitinating enzyme *Fat Facets* [49] prompted genetic suppressor screens to identify potential PHR ubiquitination targets. A MAP kinase kinase kinase (MAP3K), called Dual Leucine zipper-bearing Kinase 1 (DLK-1) in worms and Wallenda in flies, was one of the first suppressors identified [16, 48] (Fig. 1a and b). Biochemical and transgenic results indicated that RPM-1 and Highwire ubiquitinate DLK-1/Wallenda and target it for degradation by the proteasome [13, 16, 48]. Importantly, RPM-1 and Highwire function through DLK-1/Wallenda to regulate both synapse formation and axon termination [19, 40]. In mammals, Phr1 regulates Dlk ubiquitination and protein stability in dorsal root ganglion (DRG) neurons [50] (Fig. 1c). This is consistent with Phr1 restricting Dlk to axonal growth cones of DRG neurons [11]. However, Phr1 does not function through Dlk in all neuronal contexts. The dramatic defects in axon guidance in the internal capsule, the corpus callosum, and the anterior commissure of *Phr1*^Δ*8,9*^ mice are not suppressed by loss of function in *Dlk*, nor are Dlk levels globally upregulated in the brains of *Phr1*^Δ*8,9*^ mice [10, 51]. Thus, a combination of genetic and biochemical experiments across species have shown that PHR proteins function, in part, by ubiquitination and negative regulation of DLK-1/Wallenda.

Inhibition of DLK-1/Wallenda by the PHR proteins impacts the function of multiple MAP kinase pathways. RPM-1 functions in both synapse formation and axon termination by regulating a kinase cascade composed of DLK-1 (MAP3K), MKK-4 (MAP2K), PMK-3 (p38 MAP kinase), MAK-2 (MAPKAP), and the transcription factor CEBP-1 [16, 52] (Fig. 1a). Like RPM-1, several components of the DLK-1 pathway are localized to presynaptic terminals, and the DLK-1 pathway regulates local translation of CEBP-1 mRNA in the axon. This supports the intriguing possibility that RPM-1 inhibition of DLK-1 could impact both local signaling activity at the presynaptic terminal, and gene transcription.

*rpm-1* suppressor screens also yielded mutations in *uev-3*, *ess-2* and *supr-1* [46, 53]. UEV-3 is an E2 ubiquitin conjugating variant that binds to and positively regulates p38 MAPK function. It is unclear how UEV-3 regulates p38, but UEV-3 could regulate p38 target specificity, or facilitate preferential activation of p38 by upstream MAP2Ks. Alternatively, UEV-3 might regulate p38 localization, similar to the homolog UEV-1 that regulates glutamate receptor trafficking [54]. ESS-2 is orthologous to mammalian DGCR14/ES2, and regulates splicing of *dlk-1* mRNA [46]. SUPR-1 has no known function, but localizes to the nucleus suggesting a possible role in gene transcription.

Initial studies in Drosophila showed that Highwire inhibition of Wallenda/Dlk impacted the JNK ortholog, Basket, and the transcription factor c-Fos [48]. While these initial studies suggested that different MAP kinase pathways might be regulated by RPM-1 and Highwire, this was not the case as subsequent genetic analysis indicated that RPM-1 regulates a second MAPK pathway composed of the MAP3K MLK-1, the MAP2K MEK-1 (MKK7 ortholog) and the JNK isoform KGB-1 [46, 55, 56] (Fig. 1a). Notably, the DLK-1 pathway plays a primary role in regulating synapse and axon development in worms, while the MLK-1 pathway plays a secondary role. Similarly, Highwire also regulates two p38 MAPK isoforms [57]. RPM-1, Highwire and these downstream MAP kinase pathways function cell autonomously in the presynaptic neuron suggesting these are intracellular signaling pathways that control axon termination and synapse formation in invertebrate motor and sensory neurons.

The axonal growth cones of motor and sensory neurons explanted from *Phr1*^*Mag*^ mutant mice have defects in microtubule organization, which are suppressed by inhibitors of p38 MAPK [11]. This suggests that PHR proteins are conserved regulators of p38 MAP kinase signaling. Mammalian Dlk also regulates MKK7 and JNK to control neurite extension [58]. Overall, these findings argue that the PHR proteins function as conserved inhibitors of MAP3Ks that impact JNK and p38 MAP kinase signaling in the developing nervous system (Fig. 1).

The ubiquitin ligase activity of PHR proteins does not solely regulate MAP kinase cascades. The Tuberin Sclerosis Complex (TSC), composed of TSC1/Hamartin and TSC2/Tuberin, is also regulated by Phr1 and Highwire (Fig. 1b and c). A yeast two-hybrid screen for TSC2 binding proteins identified a C-terminal fragment of Phr1, and binding of Phr1 to TSC2 was confirmed from rat brain lysate [43]. Transgenic experiments in flies provided in vivo evidence that Highwire is a conserved negative regulator of TSC2 [43]. Consistent with this, the brains of *esrom* mutant fish have increased levels of phosphorylated TSC2 [8, 34]. Further, Phr1 regulates ubiquitination and degradation of TSC2 in cultured mammalian neurons, which results in inhibition of the TSC2/TSC1 complex and activation of Rheb, mTORC1 and S6 kinase [59, 60] (Fig. 1c). Consistent with in vitro findings, S6 kinase activity is reduced in the brains of *Phr1*^*Mag*^ and *Phr1*^Δ*8,9*^ mice, which have elevated TSC [51]. The nature of the genetic lesions in *Phr1*^*Mag*^ and *Phr1*^Δ*8,9*^ mice suggest that Phr1 regulates mTORC1 signaling through inhibition of TSC, and unknown ubiquitin ligase-independent mechanisms. While Phr1 regulates axon guidance in the corpus callosum, the anterior commissure, and the internal capsule, analysis of compound heterozygous *Phr1*^*Mag*^*/Phr1*^Δ*8,9*^ mice suggests that increased TSC levels and reduced mTORC1 signaling in these animals specifically impacts axon guidance in the corpus callosum [51]. Thus, inhibition of TSC and activation of mTORC1 signaling is responsible for some, but not all axon guidance defects caused by *Phr1* (lf).

Despite this important progress, findings in flies urge caution as genetic relationships between *highwire*, *TSC*, and *mTOR* are not entirely straightforward. For example, genetically altering *highwire* or *TSC* function results in abnormal synapse formation in motor neurons, which suggests these players have related functions in vivo [61]. However, while one would expect *highwire* (lf) to yield similar phenotypes to TSC overexpression, this does not occur. NMJs are abnormally small and motor neuron branches are longer and more extensive in *highwire* mutants than normal. In contrast, transgenic overexpression of TSC1 and TSC2 results in reduced numbers of NMJs and shorter axon branches. Unexpected genetic outcomes could reflect differences in neuronal context that impact the relationship between PHR proteins and TSC/Rheb/mTOR signaling, feedback loops within the TSC/Rheb/mTOR pathway, or compensation by other mechanisms that regulate activation of mTOR. Further enhancer and suppressor analysis with the PHR proteins and the TSC/Rheb/mTOR pathway in different neuronal contexts might be helpful in resolving these issues. Nonetheless, findings in flies and mice show that an important mechanism of PHR protein function is inhibition of TSC.

Genetic results are consistent with the co-Smad Medea being a potential ubiquitination target of Highwire. A yeast two-hybrid screen with a fragment of Highwire that is near the middle of the full-length protein sequence identified Medea as a Highwire binding protein [62]. Synapse formation defects in the NMJs of *highwire* mutants are suppressed by *medea* (lf) [62]. Medea regulates synapse growth by functioning in a pathway with TGFβ, the Wit BMP receptor, and the Smad transcription factor Mad. However, we note that the relationship between Highwire and the TGFβ/Medea pathway remains somewhat controversial as *wit* loss of function only partially suppresses synaptic defects caused by *highwire* (lf), and *highwire* mutants do not lead to changes in phospho-Mad levels [48]. Therefore, it remains a distinct possibility that the TGFβ/Medea pathway might function parallel to Highwire. Resolving these issues is likely to require further genetic and biochemical experiments in flies and other systems.

#### PHR proteins function as non-canonical SCF ubiquitin ligase complexes

PHR ubiquitination of Dlk is mediated by a conserved biochemical mechanism. Initially, RPM-1 was found to function in an SCF complex that includes the F-box protein FSN-1, the SKR-1 Skp protein, and the CUL-1 Cullin [63] (Fig. 1a). FSN-1 functions as the target recognition module of the complex, and RPM-1 as the catalytic E3 ubiquitin ligase. *fsn-1* functions in the same pathway as *rpm-1* to regulate synapse formation and axon termination [19, 63]. Similar to RPM-1, FSN-1 is localized to the perisynaptic region of the presynaptic terminal, and functions cell autonomously in the motor neurons to regulate synapse formation [63]. Defects caused by *fsn-1* (lf) are suppressed by *dlk-1* consistent with DLK-1 being a ubiquitination target of FSN-1 [56, 64]. The receptor tyrosine kinase ALK-1/Alk has also been implicated as a potential target of FSN-1, but this has not been followed up or analyzed in other systems [63].

PHR proteins in mammals and flies bind to the orthologs of FSN-1 called Fbxo45 and DFsn, respectively [65, 66]. *DFsn* functions in the same pathway as *highwire* to regulate synapse formation, and is suppressed by *Wallenda/Dlk*. Similar to findings in worms, synapse formation defects are not as severe in *DFsn* mutants as *highwire* mutants indicating DFsn is not the sole mediator of Highwire function. Thus, complementary results from worms and flies indicate that PHR proteins function through FSN-1 to mediate degradation of DLK-1.

*Fbxo45*^*−/−*^ mice have many phenotypic similarities with mice lacking *Phr1* function [9–11, 66]. Motor neurons have defective NMJ formation, phrenic innervation of the diaphragm is abnormal, and sensory neurons have axon termination defects in the periphery. In the brain, axon guidance is impaired. Finally, *Fbxo45*^*−/−*^ mice die shortly after birth due to respiratory distress. Notably, not all axon guidance defects in the brains of *Phr1*^Δ*8,9*^ mice are present in *Fbxo45*^*−/−*^ animals, which suggests Phr1 functions through mechanisms that are independent of Fbxo45. Consistent with common phenotypic outcomes, Fbxo45 binds to Phr1, is expressed in the nervous system, and localizes to the synapse similar to Phr1 [66, 67]. Thus, Fbxo45 is a conserved mechanism of PHR protein function. While PHR proteins function through orthologs of Fbxo45 to regulate Dlk in worms and flies, it remains unclear if Fbxo45 targets Dlk in mammals. Fbxo45 is unlikely to be the mechanism by which Phr1 ubiquitinates TSC2 for two reasons. First, *Phr1*^Δ*8,9*^ and *Phr1*^*Mag*^ mice, but not *Fbxo45*^*−/−*^ mice, have increased amounts of TSC2 [51]. Second, overexpression of Phr1 in 293 cells, but not overexpression of Fbxo45, increases mTOR activity.

The composition of SCF complexes formed by PHR proteins differs across species. As noted earlier, *C. elegans* RPM-1 is in a complex with FSN-1, SKR-1 and CUL-1 [63]. In contrast, proteomic and biochemical analysis indicate that Highwire and Phr1 form conserved, non-canonical SCF complexes containing SkpA and DFsn in flies, and Skp1 and Fbxo45 in mammals [66, 68]. Because Cullins are normally adaptors that bind to all components of a canonical SCF complex [69], it is highly unlikely proteomic screens with Highwire would identify DFsn and SkpA, but fail to detect a Cullin if one existed in this complex. Further, mutation of a single residue in the F-box domain of Fbxo45 explains the absence of a Cullin in the non-canonical Phr1/Skp1/Fbxo45 complex [66]. These findings indicate that PHR proteins generally form non-canonical ubiquitin ligase complexes that use the F-box protein Fbxo45 as a conserved target recognition module.

The enormous size of the PHR proteins (which are larger than 400 kDa) limited structure-function analysis for sometime. Initial progress came with the discovery that Fbxo45 binds to the same large fragment of Phr1 that binds Myc [66]. Subsequent biochemistry mapped binding of FSN-1 to a smaller, conserved domain in RPM-1, referred to as FSN-1 binding domain 1 (FBD1) [70]. FBD1 corresponds entirely to the conserved region of Phr1 that binds to Fbxo45, and all conserved motifs in FBD1 are required for binding to FSN-1. Therefore, FBD1 is likely to be a conserved structural mechanism that mediates PHR binding to F-box proteins. Transgenic overexpression of FBD1 results in genetic interactions and axon termination defects similar to *fsn-1* (lf). As a result, recombinantly expressed FBD1 is referred to as the RPM-1/FSN-1 complex inhibitory peptide (RIP). RIP is the first inhibitor of PHR protein function with a known biochemical mechanism of action and in vivo efficacy. Future structure-function analysis will be necessary to test two questions: 1) Does FBD1 function as a conserved site of interaction between Phr1 and Fbxo45? 2) Does FBD1 mediate direct binding to Fbxo45, or mediate direct binding to Skp1 and recruitment of Fbxo45?

#### Beyond ubiquitin ligase activity: the PHR proteins as signaling hubs

Central to the idea that PHR proteins functions as intracellular signaling hubs is evidence that PHR proteins are not solely ubiquitin ligases. Proteomic screens with *C. elegans* RPM-1 and Drosophila Highwire have revealed several proteins that bind to PHR proteins, mediate PHR signaling, and are unlikely to be targets of PHR ubiquitin ligase activity (Fig. 1a and b).

The first proteomic screen with a PHR protein was performed with *C. elegans* RPM-1 [19]. This was one of the earliest proteomic screens performed in *C. elegans* using a protein expressed exclusively in the nervous system, and identified numerous functional RPM-1 binding proteins. The first of these was Gut Granule Loss 4 (GLO-4), a putative Rab GEF referred to as Claret in flies [19, 71]. Genetic enhancer analysis revealed that *glo-4* and *fsn-1* function in parallel pathways with each mediating a portion of *rpm-1* function in synapse formation and axon termination. RPM-1 positively regulates GLO-4 as part of a pathway that includes the Rab GTPase GLO-1 and APM-3/AP3. GLO-4 colocalizes with RPM-1 at the mature axon tip and in presynaptic terminals at the perisynaptic zone, but RPM-1 is not required for GLO-4 localization. GLO-4 specifically regulates the RAB-7 late endosomal compartment at presynaptic terminals. Thus, RPM-1 is likely to function locally at the axon tip and the presynaptic terminal via the GLO-4/GLO-1/APM-3 pathway to regulate late endosome trafficking or lysosome biogenesis. Unlike *highwire* mutants, *claret* mutants do not have axon termination defects in larval C4da sensory neurons, or giant fiber pruning defects [40] (R. Murphey, personal communication). However, because *fsn-1* (lf) was needed as a sensitizing genetic background to fully reveal the role of *glo-4* in axon and synapse development in worms, results from flies do not conclusively rule out the possibility that GLO-4 is a conserved mechanism of PHR protein function. Similar lines of thinking apply to GLO-1, which has conserved orthologs in flies (Lightoid) and in mammals (Rab32, Rab38, and Rab7L1).

This proteomic screen for RPM-1 binding proteins identified several other proteins that mediate a portion of RPM-1 function including: the microtubule binding protein RNA Export 1 (RAE-1) [72], the PPM/PP2C family phosphatase PPM-2 [64], and the Nesprin ANC-1 [23] (Fig. 1a). Importantly, Rae1 was simultaneously isolated in a proteomic screen for Highwire binding proteins [73]. In yeast, RAE-1 regulates nuclear export of RNA via binding to the nucleoporin Nup98 [74, 75]. In metazoans, RAE-1 regulates microtubule stability and spindle assembly in mitotic cells, and has a more limited or no role in RNA export [76–80]. A single domain in RPM-1 and Phr1 is sufficient to mediate binding to RAE-1, and this interaction requires a small conserved motif in the PHR proteins [72]. Mutation of this motif reduces binding of RAE-1 to RPM-1 in vivo in neurons. Genetic findings in flies and worms show that *rae-1* functions in the same pathway as *highwire* and *rpm-1* to regulate synapse formation and axon termination [72, 73]. This is consistent with RAE-1 colocalizing with RPM-1 at the presynaptic terminal. Despite these similar genetic and biochemical findings, differences exist in the relationship between PHR proteins and RAE-1. In the mechanosensory neurons of worms, RAE-1 regulates axon termination by functioning downstream of RPM-1 [72]. In contrast, Rae1 protects Drosophila Highwire from degradation by autophagy suggesting Rae1 can function upstream of Highwire [73]. These differences might be explained by autophagy mediating feedback between Rae1 and the PHR proteins. Alternatively, the relationship between PHR proteins and Rae1 might differ with neuronal context. Nonetheless, a combination of biochemical and genetic results across species indicates that RAE-1 is a conserved mediator of PHR protein function.

**Fig. 2:**
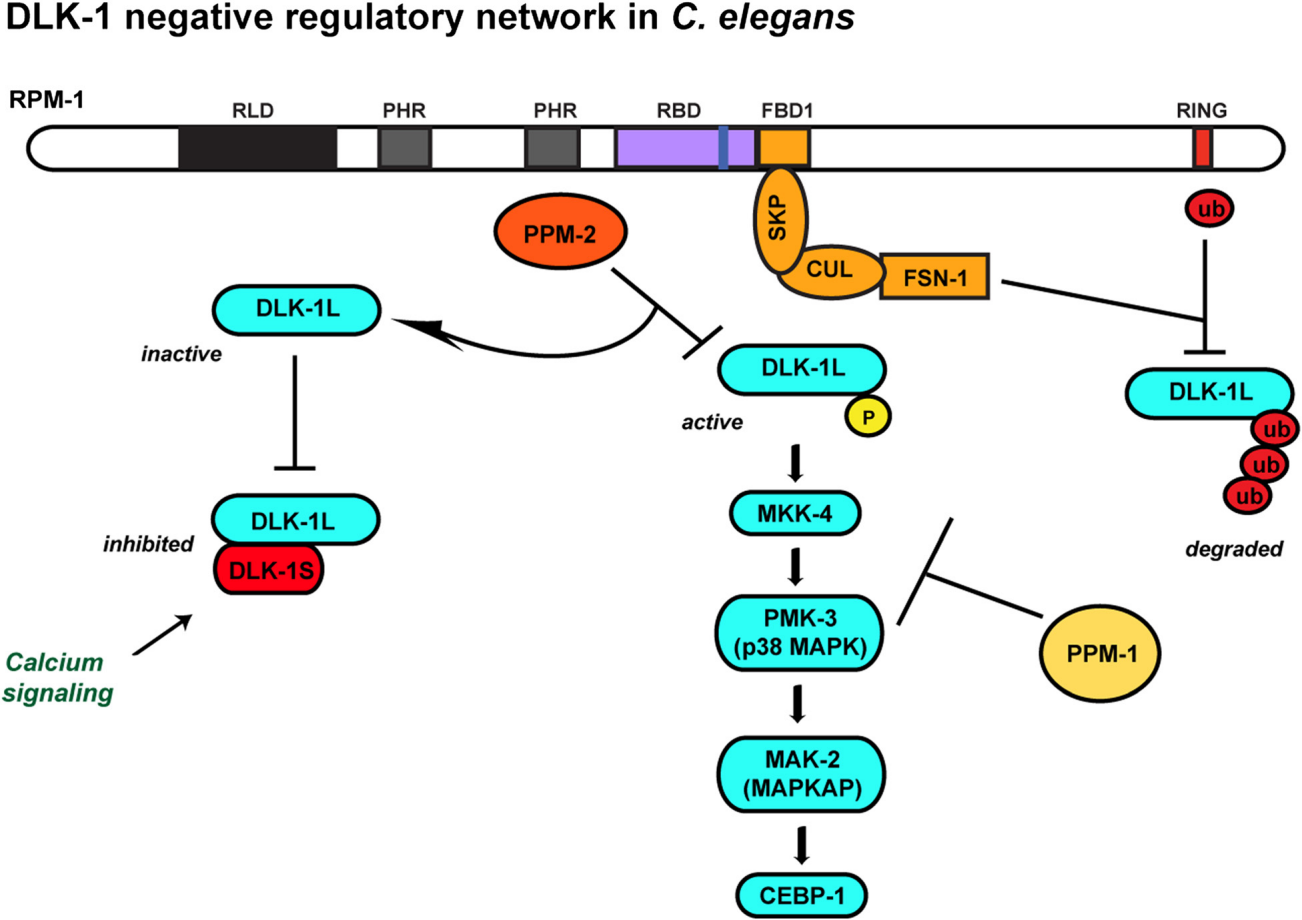
A negative regulatory network inhibits the DLK-1 pathway in *C. elegans*. Shown are the negative regulatory mechanisms that function as a network to restrain the activity of the DLK-1 MAP kinase pathway in the motor neurons and mechanosensory neurons of *C. elegans*. RPM-1 binds to PPM-2, and PPM-2 directly dephosphorylates and inhibits DLK-1 L. DLK-1S heterodimerizes with and inhibits DLK-1 L. RPM-1 and FSN-1 function as a complex to ubiquitinate DLK-1 L and target it for degradation. Finally, PPM-1 inhibits the DLK-1 pathway, most likely by dephosphorylating and inhibiting PMK-3 or MKK-4

Identification of the PPM-2 phosphatase as an RPM-1 binding protein led to the first evidence that PHR proteins employ multiple, independent mechanisms to regulate a single protein target, the DLK-1 MAP3K [64] (Fig. 2). RPM-1 inhibits DLK-1 activation via PPM-2, and regulates DLK-1 stability via FSN-1. This raises interesting implications for the spatial and temporal control of DLK-1 during development. For example, RPM-1 might function through PPM-2 to inhibit activation of DLK-1 locally at presynaptic terminals or terminating axon tips, and inhibit DLK-1 activity more globally over longer developmental time frames by regulating DLK-1 ubiquitination and degradation.

In worms, there are two isoforms of DLK-1, a full length isoform called DLK-1L and a short, inhibitory isoform called DLK-1S [81] (Fig. 2). Dephosphorylation of two serine residues in a C-terminal hexapeptide motif in DLK-1L stabilizes binding to DLK-1S resulting in formation of inactive DLK-1L/DLK-1S heterodimer. It is unclear if dephosphorylation of one or both of the serine residues in the DLK-1L hexapeptide causes heterodimerization. Calcium signaling also regulates DLK-1S inhibition of DLK-1L. PPM-2 inhibits DLK-1L by specifically dephosphorylating a single serine residue, S874, in the hexapeptide [64]. Thus, both DLK-1S and PPM-2 act on the same motif to inhibit DLK-1L. However, it remains unknown if PPM-2 directly inactivates DLK-1L independent of DLK-1S, or if PPM-2 dephosphorylation of DLK-1L is required for binding of DLK-1S. Despite the importance of the hexapeptide for regulation of DLK-1 in worms, this motif is not conserved in Drosophila or mammalian Dlk/MAP3K12 [81, 82]. However, the hexapeptide is conserved in Lzk/MAP3K13, which is highly homologous to Dlk. MAP3K13 can complement DLK-1 function in worm neurons [81], and MAP3K13 functions in neurite outgrowth mediated by the Nogo receptor [83]. These functional observations and evidence that MAP3K13 binds to Dlk [84] should prompt further exploration of MAP3K13 function in the developing nervous system. Conservation of PPM-2 in flies and the protochordate *Ciona intestinalis*, as well as conservation of the hexapeptide site where PPM-2 acts in MAP3K13 suggest that PPM-2 is likely to be a conserved mechanism of regulating MAP3K signaling.

The DLK-1 pathway is inhibited by a second phosphatase in the PPM/PP2C family, PPM-1, which is orthologous to mammalian PPM1α/PP2Cα and PPM1β/PP2Cβ [85] (Fig. 2). While PPM-2 specifically inhibits DLK-1 [56, 64], PPM-1 is a broad negative regulator of both the DLK-1 and MLK-1 pathways [56, 85]. Genetic results have hinted at the possibility that PPM-1 acts on the p38 MAPK PMK-3 in the DLK-1 pathway, but biochemistry from cultured cells leaves open the possibility that PPM-1 could inhibit MAP2Ks or MAPKs in the DLK-1 and MLK-1 pathways. Unlike PPM-2, there is no evidence indicating that PPM-1 binds to RPM-1. Thus, a network of negative regulatory mechanisms restricts DLK-1 in the neurons of *C. elegans*.

A genetic screen for NMJ morphology in fly motor neurons identified the actin and microtubule binding protein Spectraplakin as a negative regulator of Dlk signaling [82]. The molecular basis of how Spectraplakin inhibits Dlk signaling remains unclear. However, TCP1 a chaperone complex that mediates folding of actin and tubulin also regulates Dlk signaling suggesting that activation of Dlk is affected by cytoskeletal polymerization and/or stability.

The last RPM-1 binding protein identified via proteomics was the Nesprin ANC-1 [23] (Fig. 1a). *C. elegans* has a single gigantic isoform of ANC-1 (greater than 800 kDa) that is orthologous to MSP-300/Nesprin in flies, and Nesprin-1 and Nesprin-2 in mammals [86]. Loss of function in *anc-1* does not result in dramatic defects in synapse formation or axon termination, but enhancer effects were observed with an *fsn-1* (lf) background [23]. Similar to all other RPM-1 binding proteins identified by proteomics, loss of function in *anc-1* does not suppress axon termination or synapse formation defects caused by *rpm-1* (lf). These biochemical and genetic results are consistent with ANC-1 being positively regulated by RPM-1, as opposed to being ubiquitinated and degraded by RPM-1. RPM-1 and ANC-1 function in a pathway with the β-catenin BAR-1 and the transcription factor TCF/POP-1. Thus, the RPM-1/ANC-1/β-catenin/POP-1 pathway is likely to regulate axon and synapse development by impacting gene transcription. This is consistent with ANC-1 functioning at the nuclear envelope to regulate β-catenin, most likely by antagonizing EMR-1/Emerin, a known regulator of β-catenin export. Further, a combination of canonical Wnt signaling and non-canonical RPM-1/ANC-1 complex function is likely to converge on β-catenin and regulate axon termination.

Taken as a whole, several important themes emerge from these in vivo proteomic and genetic studies on RPM-1. 1) All RPM-1 binding proteins identified to date mediate a portion of RPM-1 function. Therefore, simultaneous loss of function in multiple pathways that function downstream of RPM-1 is required to cause the severe axon termination and synapse formation defects observed in *rpm-1* null animals. 2) RPM-1 both positively and negatively regulates downstream signaling pathways. 3) All RPM-1 binding proteins and downstream pathways that are known regulate both axon termination and synapse formation, rather than pathways having preferential effects on a particular developmental outcome. 4) All RPM-1 binding proteins and downstream pathways function cell autonomously to regulate axon termination and synapse formation. 5) RPM-1 is likely to affect both local signaling in the axon or presynaptic terminal, as well as impact gene transcription. 6) Finally, RPM-1 employs multiple, independent mechanisms to regulate a single target molecule, DLK-1. These findings suggest that RPM-1 is an intracellular signaling hub that uses an array of sophisticated mechanisms to control axon and synapse development. Whether this definitively emerges as an overarching theme for how all PHR proteins function will require further investigation in fly and vertebrate model systems. Importantly, identification of upstream signals that regulate PHR protein function in vivo will be essential to more definitively establish the PHR proteins as intracellular signaling hubs.

#### The conserved RCC1-like domain of PHR proteins has several biochemical activities

In vitro approaches have identified several Phr1 biochemical activities that could affect intracellular signaling and gene transcription. These findings further support our proposition that the PHR proteins function as intracellular signaling hubs (Fig. 1c, right panel). However, it remains largely unknown if PHR proteins function through these biochemical activities to regulate neuronal development in vivo.

The Bishop lab first identified Phr1 (also referred to as Pam) in a screen for Myc binding proteins [4]. The Myc binding domain (MBD) of Phr1 encompasses an N-terminal region that is conserved in RPM-1 and Highwire, and a C-terminal non-conserved region [70]. The conserved N-terminal region corresponds with FBD1, the domain in RPM-1 that mediates binding to FSN-1. Because all conserved motifs in FBD1 are required for binding to FSN-1 [70] and proteomic screens for proteins that bind to RPM-1 and Highwire have not identified Myc [19, 23, 64, 73], it is likely that Myc binds to the portion of the MBD that is not conserved in RPM-1 or Highwire.

The RCC1-like domain (RLD) of Phr1 binds to several molecules. Yeast two-hybrid screens identified different regions of the RLD that bind to type V adenylate cyclase [87], and the potassium-chloride co-transporter KCC2 [88]. The RLD inhibits type II and V adenylate cyclases in vitro, which is consistent with anti-sense oligonucleotides against Phr1 increasing adenylate cyclase activity and cAMP levels in cultured cells and neurons of the spinal cord [87, 89, 90]. Coimmunprecipitation verified binding of KCC2 to the RLD, and the RLD stimulates transporter activity in cultured cells [88]. Interestingly, the RLD acts as a Guanine Nucleotide Exchange Factor (GEF) for Rheb and Ran in vitro, which is consistent with homology between the RLD of Phr1 and the Ran GEF RCC1 [60, 91]. In cultured DRG neurons, loss of Phr1 results in increased Ran localization in the nucleus, which is consistent with Phr1 functioning as a Ran GEF [91]. SUMOylated RanGAP1, which inactivates Ran, also binds to Phr1 and inhibits Phr1 ubiquitin ligase activity. These biochemical activities of the RLD are intriguing, and future experiments testing how Phr1 regulation of adenylate cyclase, KCC2, or Rheb impacts neuronal development and/or function in vivo is eagerly awaited.

#### PHR protein signaling impacts the microtubule cytoskeleton

Genetic, biochemical and pharmacological approaches have highlighted important links between PHR protein function and the microtubule cytoskeleton. For example, in explants of cultured sensory neurons from *Phr1*^*Mag*^ mice, axonal growth cones have abnormal morphology and disorganized microtubules, which is consistent with Phr1 binding to polymerized microtubules [11]. Application of taxol (a microtubule stabilizer) rescues these defects suggesting that Phr1 stabilizes microtubules or facilitates microtubule polymerization. Cultured cortical explants from *esrom* mutant fish also have abnormal growth cone morphology that results from microtubule defects [44]. However, application of nocodazole (a microtubule depolymerizing agent) rescues this phenotype suggesting that Phr1 inhibits microtubule assembly or positively regulates disassembly. These opposing pharmacological outcomes could reflect differences in the type of neurons analyzed, developmental timing, or the use of in vitro cell culture systems.

Two other observations link PHR protein function to the microtubule cytoskeleton. The first is that Dlk regulates microtubule dynamics in many types of neurons and cultured cells [57, 82, 92–95]. The second is that invertebrate PHR proteins bind to and function through RAE-1, a microtubule binding protein [72, 73, 96]. Conceivably, RAE-1 might mediate binding of PHR proteins to microtubules, or act as a mechanism by which PHR proteins impact microtubule dynamics. This body of work prompts two important questions. 1) How do PHR proteins integrate with known regulators of microtubule assembly and disassembly? 2) How do PHR proteins impact microtubule stability and growth cone development in vivo?

At present, very little is known about whether PHR proteins regulate the actin or intermediate filament cytoskeleton, but two observations hint at this possibility. First, Phr1 binds to polymerized F-actin in vitro, and F-actin inhibits Phr1 ubiquitin ligase activity [97]. Second, Phr1 binds to Gigaxonin, an intermediate filament binding protein [98]. This is a potentially interesting observation, not only because of the implication that PHR proteins might regulate intermediate filaments, but also because mutations in *Gigaxonin* cause giant axonal neuropathy.

### The coordinator hypothesis

Synapse formation, axon guidance, and termination of axon outgrowth are often analyzed individually, but these developmental events are temporally and spatially coordinated. For example, there is a tight temporal link between axon extension and synaptogenesis in developing fish and frog RGCs, cultured mammalian hippocampal neurons, and Drosophila motor neurons [99–106]. Impaired synaptic activity in worms, flies and fish leads to abnormal axon outgrowth and branching [100, 107–109]. Morphogens, axon guidance cues, and cell adhesion molecules function in both axon outgrowth and synapse formation [110]. This could result from repurposing extracellular cues at different times and with differing neuronal context during development, but it might also reflect the role of extracellular signals in coordinating axon outgrowth and termination with synapse formation. Examples of guidance cues regulating both axon guidance and synapse formation in a single type of neuron are exemplified by the role of UNC-6/Netrin in the RIA and AIY neurons [111], and UNC-6 and Wnt effects on the DA9 motor neuron in worms [112–114]. In adult flies, Netrin regulates short-range axon guidance, as well as electrical synapse formation and function in the giant fiber [115]. These findings suggest that coordination of synapse formation with axon outgrowth and termination is likely to be a conserved feature of nervous system development. Nonetheless, the intracellular signaling mechanisms that regulate coordination remain poorly understood.

Two groups have suggested that PHR proteins regulate transitions between axon and synapse development [2, 22]. We would take this a step further by suggesting that the PHR proteins are intracellular signaling hubs that could function to coordinate different events in axon development [64]. This is a postulate we refer to as the “coordinator hypothesis”. The coordinator hypothesis rests on several observations from different systems. 1) As discussed earlier, the PHR proteins are likely to function as intracellular signaling hubs, which both positively and negatively regulate multiple downstream pathways. PHR hub function is of central importance to the coordinator hypothesis, as sophisticated signal regulation is likely to be characteristic of a coordinator. An excellent example of sophisticated signal regulation is RPM-1 utilization of both phosphatase and ubiquitin ligase mechanisms to regulate a single molecule, DLK-1 [64]. This allows RPM-1 to potentially regulate local, fast-acting DLK-1 activity as well as long-term DLK-1 protein stability and turnover. 2) PHR proteins are functionally promiscuous with conserved roles in a range of events in axon development including: synapse formation, axon guidance, and axon termination. 3) The PHR proteins regulate multiple developmental events in the same, single neuron. For example, RPM-1 and Highwire function cell autonomously in the mechanosensory neurons and the motor neurons to regulate both synapse formation and axon termination in single cells [5–7, 20]. 4) Finally, there is evidence that PHR proteins are localized to multiple subcellular compartments within a single neuron. For example, RPM-1 is localized at the mature, terminated axon tip and the presynaptic terminal in mechanosensory and motor neurons [6, 12, 20]. Low levels of RPM-1 are also present in neuronal cell bodies, and RPM-1 functions through ANC-1, which acts at the nuclear envelope [23]. Thus, RPM-1 potentially affects signaling in three subcellular compartments.

While the coordinator hypothesis is an intriguing and potentially unifying concept of PHR protein function, important questions remain regarding this postulate. For one, are PHR proteins regulated by upstream signals, in particular, extracellular signals? Two observations have hinted at this possibility. In flies and worms, PHR proteins affect signaling pathways that are regulated by extracellular cues, such as BMP and Wnt [23, 62]. Thus, PHR protein activity does integrate with signals coming from outside the cell. Second, serum stimulation of cultured DRG neurons increases nuclear localization of Phr1 [91]. The elusive nature of potential upstream cues, despite extensive genetic screens that identified both Highwire and RPM-1, suggests that multiple upstream signals are likely to regulate the PHR proteins. Another important question is whether or not PHR proteins, or molecules downstream of PHR proteins, traffic between different subcellular compartments. Finally, and most importantly, can we find direct in vivo evidence that loss of PHR protein function impairs coupling of synapse formation and axon termination. Addressing these questions will be essential in refuting or further supporting the coordinator hypothesis.

### Postdevelopmental functions of PHR proteins

PHR proteins have important functions in development of the nervous system, but PHR expression persists into adulthood. For example, endogenous and transgenic RPM-1 is detected at both the mature axon tip and the presynaptic terminals of motor and mechanosensory neurons in adults [6, 7, 20]. Transgenic Highwire is localized to presynaptic terminals at NMJs after completion of larval development [13]. Likewise, Phr1 mRNA and protein are expressed in the adult rodent brain [14, 15, 116]. Consistent with expression into adulthood, PHR proteins have important postdevelopmental functions. We already touched on Highwire regulation of long-term aversive memory in adult flies. Here, we focus on the prominent role of PHR proteins in axon degeneration.

#### Axon degeneration after injury

PHR proteins are conserved regulators of axon degeneration following traumatic injury. The first evidence for this came from genetic studies in flies, which showed that *highwire* (lf) dramatically blocks degeneration of neuromuscular junctions caused by α*-spectrin* knockdown [117, 118]. *highwire* mutants also show striking resistance to axon degeneration induced by trauma [119]. Crushing the body of a larval fly results in motor axon severing, and the severed axon segment undergoes a form of degeneration referred to as Wallerian degeneration [120]. Wallerian degeneration is vigorously blocked in *highwire* mutants, and functional synaptic connections are maintained in severed axons [119].

In vivo mouse models of Wallerian degeneration in the peripheral and central nervous system involve axotomy of the sciatic or optic nerve, respectively. In both cases, the distal axon fragment degenerates extensively within 3–5 days. In contrast, axon degeneration is dramatically halted in *Phr1*^Δ*8,9*^ mice [121]. Like flies, severed axons not only persist robustly for 5–10 days after axotomy, but synapses also remain intact for several days. Axon degeneration induced by neurotoxicity is also reduced in *Phr1*^Δ*8,9*^ DRG neurons.

**Fig. 3:**
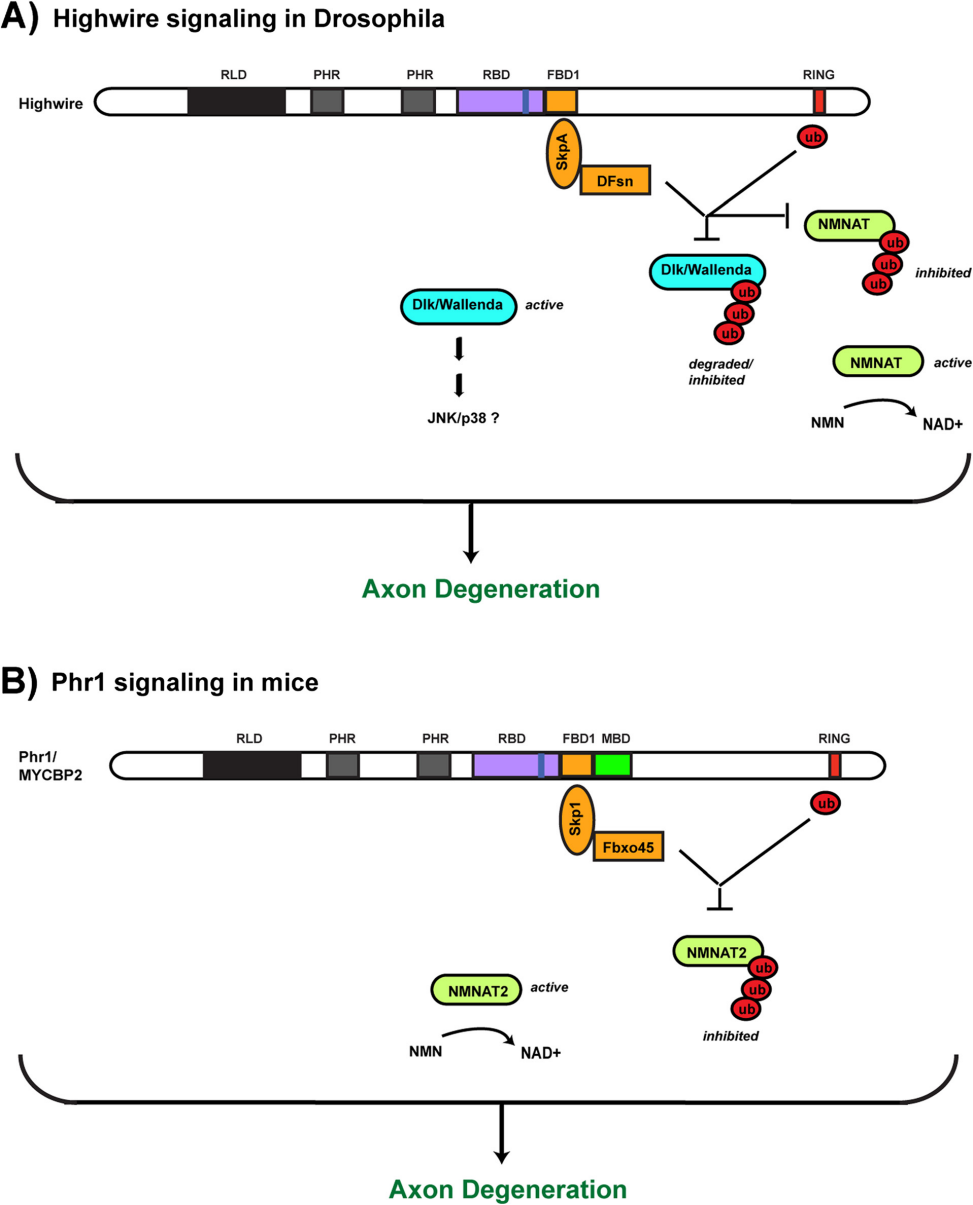
Overview of PHR protein signaling that regulates injury-induced axon degeneration in flies and mice. Shown are signaling pathways that mediate PHR protein function in axon degeneration. **a** Drosophila Highwire functions through both Dlk/Wallenda and NMNAT to regulate axon degeneration. **b** Phr1 functions through NMNAT2 to regulate axon degeneration. The MAP2K and MAPK that mediate Dlk/Wallenda function in axon degeneration remain unknown, but are likely to be either JNK or p38 MAPK based upon findings in the context of neuronal development

Highwire and Phr1 regulate axon degeneration by functioning cell autonomously in damaged neurons. In flies, Highwire regulates axon degeneration primarily by ubiquitinating and regulating levels of NMNAT, and secondarily by regulating levels of Dlk/Wallenda [119] (Fig. 3a). Mammals have multiple isoforms of NMNAT, and Phr1 functions specifically through NMNAT2 to regulate axon degeneration [121] (Fig. 3b). In mice, *Dlk* (lf) gives similar, but much more minor, effects compared to *Phr1* (lf) [122]. This suggests that Phr1 is unlikely to regulate axon degeneration via Dlk. If this were the case, we would expect *Phr1* (lf) to have similar phenotypes to Dlk overexpression not *Dlk* (lf). While it is unclear if RPM-1 regulates axon degeneration in worms, excess NMNAT does reduce axon degeneration [123]. These findings indicate that PHR proteins are conserved, central players in an actively regulated axon degeneration program.

The molecular mechanism of how PHR proteins ubiquitinate NMNAT remains unclear. However, the F-box protein Fbxo45 binds NMNAT in HEK 293 cells suggesting that Fbxo45 could mediate Phr1 ubiquitination of NMNAT [121]. If this proves to be the case, molecules that inhibit the Phr1/Fbox45 complex could be potent inhibitors of axon degeneration. Interestingly, increased NMNAT function is protective in several models of neurodegenerative disease including: glaucoma, axonopathies, Parkinson’s disease, and Tauopathy [120]. Thus, inhibitors of the Phr1/Fbxo45 complex could prove potentially useful in slowing the progression of several neurodegenerative diseases. Recent work in worms identified RIP as an in vivo inhibitor of the RPM-1/FSN-1 complex [70]. This could represent an important first step towards developing inhibitors of the Phr1/Fbxo45 complex.

#### Axon regeneration after injury

PHR proteins play a modest role in axon regeneration. In worms, laser axotomy of individual neurons is used to assess axon regeneration [124]. Loss of function in *rpm-1* or *fsn-1* modestly increases axon regeneration in motor neurons [55, 125]. Similarly, *highwire* (lf) accelerates axon regeneration in fly motor neurons [42]. Both RPM-1 and Highwire function through DLK-1/Wallenda to regulate regeneration [52, 125]. Young worms regenerate with much greater capacity than older worms, and the improvements in axon regeneration that come with increased DLK-1 function are noticeably more pronounced in older animals [125]. Therefore, loss of function in *rpm-1* or other molecules that lead to increased DLK-1 activity might give more prominent improvements in regeneration in older animals where regeneration is more limited. To our knowledge, it is unknown if loss of Phr1 results in improved axon regeneration in mammals, but Dlk is required for axon regeneration in mammals similar to invertebrates [126, 127]. These conserved functional outcomes in different model systems suggest that PHR proteins are likely to influence an important molecular choice point that regulates the balance between axon regeneration and axon degeneration.

### Emerging genetic links between the PHR protein signaling network and neurodevelopmental disorders

To our knowledge, direct genetic links between human *MYCBP2/PAM* and neurodevelopmental disorders have not been found. This includes a study that examined the *MYCBP2* locus in 300 autism patients [128]. However, there are numerous genetic links between signaling pathways that are regulated by PHR proteins and neurodevelopmental disorders.

The most prominent of these links is Phr1 regulation of TSC [43, 51, 59]. Mutations in *TSC1* or *TSC2* cause tuberous sclerosis, which results in non-malignant tumors in the brain, autistic behavior, intellectual disability, and seizures [129, 130]. Many of these symptoms potentially result from brain tumors, but might also stem from increased mTOR signaling which leads to defects in synapse formation, function, or plasticity. Interestingly, one disease-associated mutation in *TSC2* causes increased ubiquitination by Phr1 [59]. As a result, enhanced degradation of TSC2 by Phr1 might directly impact a subset of patients with tuberous sclerosis.

As discussed earlier, Fbxo45 functions in a ubiquitin ligase complex with Phr1. *Fbxo45* is one of several genes deleted in 3q29 microdeletion syndrome, which results in autism, intellectual disability, and schizophrenia [131–135]. *Fbxo45* is duplicated in patients with 3q29 microduplication syndrome, which results in intellectual disability and seizures [136, 137]. 3q29 deletion and duplication regions contain several genes of neuronal relevance, but the role of *Fbxo45* in neuronal development and synaptic function suggests it could be a relevant player in these syndromes. A mutation in *Fbxo45* is also associated with schizophrenia [138]. Interestingly, microdeletions [139–141] and microduplications [142, 143] that include *Dlk/MAP3K12*, a likely Fbxo45 ubiquitination target, also result in intellectual disability and autism. Splicing of *dlk-1* in worms is regulated by DGCR14/ES2 [46]. DGCR14 is present in chromosomal deletions implicated in DiGeorge syndrome, which is characterized by autism and schizophrenia [144–148]. While these findings might be a coincidence, they could also reflect the conserved functional relationship between Fbxo45, Dlk, and Phr1.

The discovery that RPM-1 functions, in part, by binding to the Nesprin ANC-1 opens up another genetic link between PHR signaling and neurodevelopmental disorders. *C. elegans* ANC-1 is most similar to mammalian Nesprin-1 (also called Syne1). Mutations in *Nesprin-1* cause cerebellar ataxia [149–153], and are associated with autism [154, 155]. Further, *Nesprin-1* SNPs are associated with bipolar disorder [156–160] and schizophrenia [161, 162]. Collectively, these findings highlight potentially important genetic links between PHR signaling and neurodevelopmental disorders.

## Conclusions

Research over the past decade has greatly expanded our understanding of the PHR proteins on numerous fronts. 1) Mounting evidence indicates that PHR proteins both positively and negatively regulate multiple downstream signaling pathways, at times through sophisticated mechanisms. Thus, rather than solely functioning as ubiquitin ligases, the PHR proteins are most likely intracellular signaling hubs. 2) Collective results, particularly from worms, have begun to coalesce around the concept of the PHR proteins as signaling hubs that coordinate different events in neuronal development, a postulate we refer to as the coordinator hypothesis. 3) Consistent with an important and broad role in neuronal development, a striking number of genetic links between PHR signaling networks and neurodevelopmental disorders have emerged. 4) Finally, PHR proteins are important regulators of axon degeneration, which has potentially important implications regarding neurodegenerative disease. While this highlights exciting progress, much still remains to be learned about these physically enormous and molecularly rich signaling molecules.
